# A study on the influencing factors and related paths of farmer’s participation in food safety governance—based on DEMATEL-ISM-MICMAC model

**DOI:** 10.1038/s41598-023-38585-w

**Published:** 2023-07-14

**Authors:** Jie-Hui Xie, Fu-Jun Tian, Xue-Yuan Li, Yu-Qing Chen, Shi-Yi Li

**Affiliations:** 1 School of Public Administration, Fujian Agriculture and Forestry University, Fuzhou, 350002 Fujian China; 2 Rural Development Research Center, Fujian Agriculture and Forestry University, Fuzhou, 350002 Fujian China

**Keywords:** Health care, Risk factors

## Abstract

Farmers' participation in food safety governance is an important part of food safety social co-governance, and the accurate identification of its influencing factors and their related paths is of guiding significance to the scientific decision-making of food safety governance. The system of influencing factors of farmers' participation in food safety governance was constructed from four dimensions, and the influence network of each dimension was revealed by decision laboratory analysis (DEMATEL). The hierarchical structure and correlation path of influencing factors were determined by interpretive structural model (ISM), and the attributes of influencing factors were further classified by cross influence matrix multiplication (MICMAC). The results show that the influencing factors of farmers' participation in food safety governance can be divided into seven levels, among which the level of education and the status of village cadres are the fundamental characteristic factors. The degree of rural informatization, the intensity of government supervision, the promotion of village committees, the response of the government and the degree of disclosure of government information are the deep core factors, and risk cognition, political trust and family eating habits are special factors. Taking the importance and attribute status of farmers' participation in food safety governance into decision-making considerations is of great significance to improve the efficiency of food safety governance.

## Introduction

As an ancient Chinese wisdom saying highlights, “Among eight most important governance issues, food stands atop”. General Secretary Xi Jinping emphasizes that, "Whether or not we can give the people a satisfactory account of our food safety is a major test of our ability to govern." The 14th Five-Year Plan for National Economic and Social Development of the People's Republic of China and the outline of the 2035 Visionary Goals proposed the "deeply implement the food safety strategy", which has elevated food safety to an important strategic position in China, with major livelihood issues and national public safety at stake. China Health Statistics Yearbook by the end of 2020: 7073 outbreaks of foodborne diseases reported nationwide, an increase of 683 over the previous year; According to the 2022 Global Food Safety Index report (Global Food Security Index) released by the British Economist Intelligence Unit, China's food quality and safety assurance capability ranked 43th out of 107 countries, still lagging behind developed countries. The current situation of food safety governance in China is still not optimistic, especially in the rural field, there are a series of "double failures" between the market and the government, such as economic backwardness, asymmetric market information, unfair distribution of regulatory resources, non-standard supply system and so on^1^.

With the continuous improvement of social governance, all sectors of society are also aware of the phenomenon of "government failure" in food safety supervision, and relying solely on government departments cannot effectively prevent food safety risks. Since the reform and opening up, China's food safety supervision mode has followed the logic of development from adversarial supervision to cooperative supervision, and social co governance has become a new model to focus on building^2^. As an important part of public security governance and social governance system, food safety governance is related to national security and social stability. Although China is in the stage of rapid urbanization, the rural population still accounts for 36%. To improve the current situation of food safety governance in China, it is urgent to focus on the weak rural areas of food safety governance and explore the influencing factors of farmers' participation in food safety governance, so as to respond to the practical needs of improving the level of rural food safety governance. Therefore, this study quantitatively analyzes the influencing factors of farmers' participation in food safety governance and the relationship among them from four dimensions and 20 indicators including family characteristics, participants, participation process and participation environment with DEMATEL method, further analyzes the overall hierarchical structure and correlation path of the influencing factors of farmers' participation in food safety governance through ISM model, and divides the attribute of factors through MICMAC analysis method, Find out the key factors that affect farmers' participation in food safety governance, in order to provide scientific decision-making basis for promoting farmers' participation in food safety governance.

## Review of related literature

The international research on public participation in food safety governance started early. Henson and others first put forward the co-governance model in the field of food safety, believing that the efficiency of food safety governance can be improved through the cooperation of public and private sectors^3^. On the basis of making it clear that food safety governance needs co-governance, the research on multiple subjects, especially the public participation model, participation effectiveness and its influencing factors is also gradually enriched. Based on the fact that consumers' confidence was frustrated by frequent food safety incidents in Europe, Cope et al. compared expert and consumer data through internet surveys, case interviews and other methods, found that the effectiveness of food safety risk communication mechanisms was affected by consumers' risk perception and food safety information needs through empirical studies. It mainly includes consumers' individual preferences, differences in information needs, social, historical and cultural environment, it is proposed that food risk governance should enhance communication with skateholders in a transparent and accountable manner^4^, where multiple survey methods are used and the comparison of the differences in the groups interviewed makes the article persuasive, with the conclusion that The ability to identify the attributes that food safety risk communication messages should have is a realistic guide to the construction and improvement of food safety risk communication mechanisms. Truong et al. believe that food quality certification has been widely promoted in solving food safety issues that consumers are increasingly concerned about, and that the question that needs to be addressed is whether and how the trustworthiness of food actors (e.g. growers and retailers) affects consumers' trust in food certification and their food choices, based on the principles of a typical qualitative study, collected data from in-depth interviews with 27 relevant participants in Vietnam and found that differences in consumers' trust in certification depended on their perceptions of the trustworthiness of the food system and its participants in providing certified food^5^.

The sudden increase in research on food safety regulation in China began with the outbreak of melamine in 2008^6^, which started later than abroad, but with the promulgation and revision of the Food Safety Law of the people's Republic of China, social co-governance has become one of the basic principles of food safety governance in China, and the research on food safety governance based on the perspective of public participation is increasing day by day. The research theme is mainly divided into the necessity of public participation and the influencing factors of public participation. The latter is carried out by combining risk governance theory, game behavior theory and planned behavior theory^7,8^. From the perspective of risk governance, public risk perception, risk communication mechanism, as well as their own experience and past experience are the main factors that affect their participation. According to the game behavior theory, the behavior decisions of stakeholders in food safety, including the public, are the result of games among various subjects. Wu Ye investigated the game and dysfunctional behaviour of government, food enterprises and consumers under food safety information asymmetry, and the behaviour of third parties in polycentric governance on the optimisation of regulation, starting from the goal of optimising food safety regulation. The study shows that the timely release of government authoritative information and the intervention and guidance of professional or highly reputable social institutions can improve the public's cognitive ability of food safety information^9^. Ma Qiaoyun developed an evolutionary game model between grassroots government and new professional farmers in the process of rural food safety governance, based on the Chinese rural context, by introducing villagers' committees and the public as third-party monitoring forces. The results demonstrate that the government should bring village committees into the supervision system and simplify the reporting process and expand reporting channels. To facilitate the participation of villagers' committees and the public in rural food safety governance^10^. The combination of the game model and the research topic is undeniably innovative, but the assumed premise of economic man leads to an oversimplification of the complex problem of shared food safety governance, ignoring the influence of internal and external factors such as psychological and environmental factors. In the theory of planned behavior, the public's behavior attitude, subjective norms and perceptual behavior control are the three main variables that determine their intention to participate in behavior. Considering the regional variability of food safety consumption risks in rural areas and the heterogeneity of rural residents' degree of concern about food safety consumption, Wang considers the regional differences in food safety consumption risks in rural areas and the heterogeneity of rural residents' concerns about food safety consumption, Wang Jianhua believes that there is a certain degree of difference between the food safety consumption attitudes, willingness, and behavior of rural residents in China. Based on empirical research data on food safety consumption of rural residents in 500 natural villages in 20 provinces in China, The global Moran'I index and local Moran'I autocorrelation index using spatial correlation testing were used to conduct in-depth analysis of rural residents' attitudes towards food safety consumption. The results showed that the main influencing factors for the differences include subjective normative effects, perceived behavioral control effects, inherent consumption habits barriers of rural consumers, incomplete construction of safe food consumption infrastructure Insufficient government supervision and certification efforts, as well as the lack of relevant policies and systems^11^. Its research creatively combines rural residents' attitudes towards food safety consumption with spatial econometric methods, and identifies multiple influencing factors for differences in rural residents' attitudes towards food safety consumption. However, further exploration has not been conducted on the correlation between these factors, which lacks guidance for policy formulation.

From existing literature, it can be found that the current quantitative research on the influencing factors of public participation in food safety governance has diverse perspectives and rich methods, which can provide certain reference significance for this study. However, there are still shortcomings in existing research: firstly, most studies focus on urban residents, but there is still a lack of research on the influencing factors of farmers' participation in food safety governance; Secondly, most studies only unilaterally consider internal factors such as subjective norms and perceived behavioral control, or external factors such as policy systems, lacking systematic consideration of internal and external factors; Finally, existing quantitative studies often use LOGIT regression, structural equation models, evolutionary game models, etc., which can summarize significant influencing factors, but fail to deeply reveal the correlation paths between influencing factors and distinguish the importance and attribute positioning of influencing factors. On this basis, this article adopts the combination method of DEMATEL-ISM-MICMAC to study the influencing factors and associated pathways of farmers' participation in food safety governance, further filling the gap in existing research.

Food security is related to the national economy and people's livelihood. In terms of food quantity security, basic food security problems still exist. The growing World population and limited natural food production capacity are the root causes of the growing food security problems around the world^12^. In addition, the 2019 coronavirus epidemic has a direct impact on the food system by affecting the food supply and demand system, and by reducing purchasing power Reducing food distribution and marketing capabilities, as well as increasing healthcare workload, have had indirect impacts^13^, especially in impoverished countries with a predominantly agricultural industry, where household food security is worrying^14^. For example, during the pandemic in Ghana, due to the lockdown policy, the number of fresh food suppliers in the market has decreased, as well as the closure of restaurants, hotels, and other local restaurants, both food security and food quality safety cannot be adequately guaranteed^15^. In terms of food quality and safety, according to surveys, the abuse of food additives and the malicious addition of non edible substances, excessive residues of agricultural and veterinary drugs, bacterial and harmful microbial pollution, and heavy metal pollution are the most worrying food safety risk factors for urban and rural residents^16^.

From a research perspective, given the high importance of food safety assurance to many governments and society, it is necessary to examine it from the perspective of governance. Many factors that affect food safety, such as political, economic, social, and environmental factors, can be rooted in governance^17^. From the perspective of research subjects, both farmers based on the Chinese context and impoverished groups around the world are facing daunting food safety issues, and are in a relatively disadvantaged position in the field of public participation in food safety. Therefore, studying the influencing factors and associated pathways of farmers' participation in food safety governance has certain practical significance.

## Determination of influencing factors of farmers' participation in food safety governance

Farmers' participation in food safety governance is not only an important part of food safety social co-governance, but also a specific form of public participation in the field of food safety. In order to scientifically and reasonably determine the influencing factors of farmers' participation in food safety governance, we can take it as a system to specifically consider the internal and external environment of the system^18^. On the basis of collating, analyzing and summarizing the relevant literature and policy documents on the influencing factors of food safety governance, public participation and farmers' participation in food safety governance, combined with the results of discussion and screening by the expert group (mainly focused on public management and food safety governance). A system of 20 influencing factors including family characteristics, participants, participation process and participation environment is identified (as shown in Table 1).

**Table 1:** System of influencing factors of farmers' participation in food safety governance.

Dimension	Serial number	Factor name	Meaning	Reference source
Family characteristics	a1	Education level	Average educational level of family members	^19,20,21^
	a2	Village cadre status	Family members have held or held the post of village cadre in their village	^22^
	a3	Household income	Per capita monthly income level of family members	^19,23,24^
	a4	Family structure	Proportion of children (under 12 years old) and elderly (60 years old and above) in family members	^23,25,26^
	a5	Family eating habits	The frequency of family or family members dining out and ordering take-out food	^27^
	a6	Self-supply of food	The proportion of household produced food (grain, vegetables, meat, etc.) for household consumption in total food consumption	^28,29^
Participation subject	a7	Victim experience	Farmers' experience of suffering from food safety problems such as fake and inferior products and being damaged	^21,24,30^
	a8	Political trust	The degree of trust of farmers in county (district, city), township (town) and village cadres	^8,17,31^
	a9	Risk perception	Farmers' perception of the attributes (safety) in the food and the possible health consequences and severity of the food after consumption	^7,8,17,32^
	a10	Media attention	The exposure and continuous reporting of food safety incidents by the mass media and the guidance of the official authoritative media on food safety information	^33,34,35^
	a11	Government supervision	The government competent authority regulates and restricts the production, processing and circulation of food by formulating and promulgating relevant food safety laws and regulations and adopting administrative measures	^9,10,35^
	a12	Promotion of village committee	The village committee assists the government in the implementation of food safety public services and the promotion of farmers' participation in food safety governance	^22,36,37^
Participation process	a13	Perception of participation effectiveness	To what extent can farmers' participation affect the efficacy perception of food safety governance	^38,39^
	a14	Participation cost perception	Farmers' perception of how much time, money, energy and other costs are needed to participate in food safety governance	^40^
	a15	Government response	Timeliness and effectiveness of the government's response to farmers' opinions, suggestions, complaints and reports on food safety	^38,41^
Participation environment	a16	Participation atmosphere	More people around participate in or support food safety governance	^37,42^
	a17	Rural informatization degree	Rural informatization infrastructure construction and farmers' acceptance and use of information technology	^34,38^
	a18	Publicity of government information	Publicity of food safety information by government authorities	^38,41^
	a19	Participation channels	Diversification and perfection of channels for farmers to conduct consultation, suggestions, complaints and reports	^26,43^
	a20	Incentive mechanism	The mechanism of corresponding rewards for reporting illegal acts of food safety	^43^

## Modeling analysis of influencing factors of farmers' participation in food safety governance

### Network analysis of influencing factors of farmers' participation in food safety governance– DEMATEL

Decision laboratory analysis (Decision making trial and evaluation laboratory, DEMATEL) is a method put forward by American scholars to analyze system factors by using graph theory and matrix theory. Through the logical relationship and direct influence matrix among the elements in the system, the influence degree of each factor on other elements and the degree of influence can be calculated, thus the cause degree and center degree of each element can be calculated^44^. The advantage of this method is that it can make full use of and synthesize the knowledge and experience of experts to deal with complex system problems, and use specific numerical values to express the relationship among the factors in the system^45^. Regarding the issue of complex systems, correlation is more important than randomness and representativeness. Therefore, a total of 20 experts and scholars were invited to conduct a structured questionnaire survey. This article received funding from the Provincial Social Science Research Base. In the project research, 20 experts were invited to serve as decision advisors for this project, including university professors, research institute researchers, and public governance practice experts. The research direction is public management and food safety governance, with relevant theoretical knowledge or practical experience. Before conducting this research, The expert group has conducted on-site research and inspections in rural areas in multiple regions to understand the actual situation of farmers' participation in food safety governance, which can ensure that the questionnaire filling has a certain quality. The experts scored the pairwise influence degree of the above 20 indicators on the basis of experience and professional cognition. The scoring system is 0–4: 0 as no impact, 1 as weak impact, 2 as moderate impact, 3 as strong impact and 4 as extremely high impact. All data generated or analysed during this study are included in this published article [and its supplementary information files]. Through the reliability test of the recovered questionnaire by SPSS26.0, the overall Cronbach's α value is 0.994. In view of the consideration that the amount of matrix scoring questionnaire has some influence on the final reliability, the questionnaire data are divided into 20 dimensions according to the influencing factors, and the lowest reliability coefficient is 0.863, all of which are more than 0.8. The reliability is high.

#### Calculation of centrality and causality

The main contents are as follows:

1. Establish the direct influence matrix A. In order to eliminate individual differences, the average value of 20 valid data collected was processed, and the direct influence matrix of farmers' participation in food safety governance was obtained, in which aij represents the influence degree of factor I on factor j, while the influence of factor on itself, so all values are 0 (and), and the results are detailed in Table 2. The direct influence matrix can reflect the direct influence relationship among the factors, but in order to further explore the indirect influence relationship among the factors, it is necessary to calculate the comprehensive influence matrix (Table 3).
2. Establish canonical influence matrix B and comprehensive influence matrix K. In order to eliminate the dimensional effect, the matrix is normalized, the direct influence matrix An is divided by its row sum and column and the maximum value c, the canonical influence matrix B is obtained, and then the comprehensive influence matrix K is calculated. the results are detailed in Table 3.
  1 $$c=\mathrm{max}\left[\underset{1\le j\le n}{\mathrm{max}}\sum_{i=1}^{m}{a}_{ij},\underset{1\le i\le m}{\mathrm{max}}\sum_{j=1}^{n}{a}_{ij}\right]$$
  2 $$B=A/c$$
  3 $$K=B{(E-B)}^{-1}$$

**Table 2:** Direct influence Matrix An of factors affecting Farmers' participation in Food Safety Governance.

Variables	a1	a2	a3	a4	a5	a6	a7	a8	a9	a10	a11	a12	a13	a14	a15	a16	a17	a18	a19	a20
a1	0	2.55	2.9	1.9	2.9	2.4	2.85	2.95	3.45	2.65	2.45	2.2	2.95	2.85	2.4	2.95	2.65	2.35	2.45	2.3
a2	2.1	0	2.85	1.85	2.25	2.3	2.35	3.2	3.1	2.75	2.7	3.4	2.9	2.85	2.7	3.1	2.35	2.75	2.9	2.6
a3	2.75	2.4	0	2.15	3.15	3.05	1.8	2.2	2.75	2.2	1.8	1.8	2	2.15	1.8	1.85	2.1	1.65	1.8	1.6
a4	2.25	1.85	2.85	0	2.8	2.4	1.5	1.65	2.3	1.95	1.5	1.6	2.1	2.3	1.45	2	1.9	1.5	1.65	1.5
a5	1.2	1.15	1.55	1.1	0	2.9	2.4	1.4	2.15	1.5	1.3	1.4	1.4	1.85	1.2	1.3	1.35	1.1	1.35	1.4
a6	1.7	1.35	2.4	1.65	3.15	0	2.05	1.5	1.7	1.5	1.4	1.35	1.65	2.1	1.3	1.65	1.35	1.2	1.45	1.3
a7	1.5	1.4	1.5	1.4	3.45	3	0	2.65	3.15	2.2	2.25	2.15	2.35	2.3	2.15	2.25	1.55	2.25	2.4	1.65
a8	1.65	2.4	1.3	1.35	1.8	1.75	1.45	0	2	2.35	2.2	2.15	2.3	2.25	2.2	2.05	1.4	1.95	2	1.6
a9	1.45	1.25	1.5	1.35	2.85	2.7	2.3	2.1	0	2.45	2.25	2.35	2.15	2.25	2.15	2.2	1.7	1.8	2	1.55
a10	1.5	1.7	1.35	1.2	2.55	2.05	2	2.5	2.8	0	2.45	2.15	1.9	2.1	2.45	2.35	1.9	2.65	2.4	1.7
a11	1.25	1.85	1.35	1.15	2.15	2.15	2.55	2.75	2.3	2.65	0	2.85	2.3	2.15	2.9	2.45	2.15	3.15	2.8	2.6
a12	1.35	1.75	1.45	1.45	2.35	2.4	2.2	2.4	2.8	2.1	2.5	0	2.45	2.25	2.45	2.7	2.35	2.6	2.7	2.55
a13	1.6	1.55	1.3	1.3	2.35	1.95	1.8	2.4	2.65	2	1.8	1.85	0	2.35	2.05	2.2	1.8	1.85	1.95	2
a14	1.45	1.45	1.8	1.25	2.25	2.2	1.75	2	2.25	1.6	1.55	1.65	2.4	0	1.65	1.9	1.75	1.85	1.9	1.85
a15	1.3	1.35	1.1	1.2	2.05	1.9	1.7	2.95	2.7	2.65	2.95	2.95	2.4	2.3	0	2.65	1.95	2.8	2.4	2.1
a16	1.25	1.35	1.2	1	2.05	1.75	1.6	2.05	2.55	2	2.45	2.4	2.75	2.4	2.35	0	1.85	2.6	2.65	2
a17	1.8	1.55	1.85	1.5	2.5	1.95	2.05	2.7	2.8	2.4	2.5	2.5	2.45	2.4	2.55	2.3	0	2.9	3	2
a18	1.1	1.3	1.1	1.05	1.95	1.8	1.85	3.25	2.65	2.3	2.65	2.85	2.4	2	2.75	2.5	2.2	0	2.3	1.95
a19	1.3	1.1	1	0.9	1.8	1.7	1.65	2.6	2.35	2.25	2.35	2.05	2.7	2.4	2.45	2.4	1.9	2.15	0	2.05
a20	1.3	1.3	1.45	1	2.05	1.55	1.45	2.15	1.9	1.85	2.2	2.2	2.45	2.2	1.75	2.75	2	2.2	1.95	0

**Table 3:** Comprehensive influence matrix K of influencing factors of farmers' participation in food safety governance.

Variables	a1	a2	a3	a4	a5	a6	a7	a8	a9	a10	a11	a12	a13	a14	a15	a16	a17	a18	a19	a20
a1	0.12	0.17	0.18	0.14	0.24	0.22	0.21	0.24	0.26	0.22	0.22	0.21	0.24	0.23	0.21	0.23	0.20	0.21	0.22	0.19
a2	0.16	0.13	0.18	0.14	0.23	0.22	0.20	0.25	0.26	0.22	0.22	0.24	0.24	0.23	0.22	0.24	0.20	0.23	0.23	0.20
a3	0.15	0.15	0.11	0.13	0.22	0.20	0.16	0.19	0.21	0.18	0.17	0.18	0.19	0.19	0.17	0.18	0.16	0.17	0.18	0.15
a4	0.13	0.13	0.15	0.08	0.19	0.17	0.14	0.17	0.19	0.16	0.16	0.16	0.17	0.18	0.15	0.17	0.15	0.15	0.16	0.14
a5	0.09	0.10	0.11	0.08	0.11	0.16	0.14	0.14	0.16	0.13	0.12	0.13	0.13	0.14	0.12	0.13	0.11	0.12	0.13	0.11
a6	0.11	0.11	0.13	0.10	0.18	0.11	0.14	0.15	0.16	0.14	0.13	0.14	0.15	0.15	0.13	0.14	0.12	0.13	0.14	0.12
a7	0.13	0.13	0.13	0.11	0.22	0.20	0.13	0.20	0.22	0.18	0.18	0.18	0.19	0.19	0.18	0.19	0.15	0.18	0.19	0.15
a8	0.12	0.14	0.12	0.10	0.17	0.16	0.14	0.14	0.18	0.17	0.17	0.17	0.18	0.17	0.17	0.17	0.14	0.16	0.17	0.14
a9	0.12	0.12	0.13	0.11	0.20	0.18	0.16	0.18	0.15	0.18	0.17	0.18	0.18	0.18	0.17	0.18	0.15	0.16	0.17	0.14
a10	0.13	0.13	0.13	0.11	0.20	0.18	0.16	0.20	0.21	0.14	0.18	0.18	0.18	0.18	0.18	0.19	0.15	0.19	0.18	0.15
a11	0.13	0.14	0.14	0.11	0.20	0.19	0.18	0.21	0.21	0.20	0.15	0.20	0.20	0.20	0.20	0.20	0.17	0.21	0.20	0.18
a12	0.13	0.14	0.14	0.12	0.21	0.19	0.17	0.20	0.22	0.19	0.19	0.15	0.20	0.19	0.19	0.20	0.17	0.19	0.20	0.18
a13	0.12	0.12	0.12	0.10	0.18	0.16	0.15	0.18	0.19	0.16	0.16	0.16	0.13	0.18	0.16	0.17	0.14	0.16	0.16	0.15
a14	0.11	0.12	0.12	0.10	0.17	0.16	0.14	0.17	0.18	0.15	0.15	0.15	0.17	0.12	0.15	0.16	0.14	0.15	0.16	0.14
a15	0.13	0.13	0.13	0.11	0.19	0.18	0.16	0.21	0.21	0.19	0.20	0.20	0.19	0.19	0.14	0.20	0.16	0.19	0.19	0.16
a16	0.12	0.12	0.12	0.10	0.18	0.17	0.15	0.18	0.20	0.17	0.18	0.18	0.19	0.18	0.17	0.14	0.15	0.18	0.18	0.15
a17	0.14	0.14	0.15	0.12	0.21	0.19	0.17	0.21	0.22	0.19	0.20	0.20	0.20	0.20	0.20	0.20	0.13	0.20	0.21	0.17
a18	0.12	0.13	0.12	0.11	0.19	0.17	0.16	0.21	0.21	0.18	0.19	0.19	0.19	0.18	0.19	0.19	0.16	0.14	0.18	0.16
a19	0.12	0.12	0.11	0.10	0.17	0.16	0.15	0.19	0.19	0.17	0.17	0.17	0.19	0.18	0.17	0.18	0.15	0.17	0.13	0.15
a20	0.11	0.12	0.12	0.10	0.17	0.15	0.14	0.17	0.18	0.16	0.16	0.17	0.18	0.17	0.15	0.18	0.14	0.16	0.16	0.11

In the formula: B is the canonical influence matrix; K is the comprehensive influence matrix; E is the corresponding order unit matrix.

(3) Determine the influence degree, affected degree, center degree and cause degree of the influencing factors. The influence degree is the sum of the rows of the comprehensive influence matrix K, which indicates the comprehensive influence degree of one factor on other factors, including direct influence and indirect influence. The degree of influence indicates the degree to which a factor is affected by other factors. The greater the centrality, the greater the importance of the factor in the system. The positive result indicates that the factor has a greater influence on other factors, while a negative result indicates that the factor is more affected by other factors. The results are detailed in Table 4.

**Table 4:** Centrality and cause of factors affecting farmers' participation in food safety governance.

Variables	Influence degree (*m)*	Affected degree(*n*)	Centre degree(*c)*	Cause degree(*r)*
a1	4.17	2.49	6.66	1.69
a2	4.25	2.57	6.82	1.69
a3	3.44	2.62	6.06	0.81
a4	3.11	2.17	5.28	0.94
a5	2.44	3.86	6.30	− 1.42
a6	2.67	3.51	6.17	− 0.84
a7	3.43	3.15	6.58	0.29
a8	3.06	3.79	6.85	− 0.72
a9	3.20	4.01	7.21	− 0.82
a10	3.33	3.46	6.79	− 0.14
a11	3.63	3.47	7.10	0.17
a12	3.57	3.51	7.08	0.06
a13	3.08	3.67	6.75	− 0.59
a14	2.89	3.63	6.52	− 0.74
a15	3.46	3.43	6.89	0.03
a16	3.20	3.63	6.83	− 0.43
a17	3.65	3.03	6.68	0.62
a18	3.35	3.47	6.81	− 0.12
a19	3.11	3.53	6.63	− 0.42
a20	3.00	3.06	6.06	− 0.06

#### Analysis of the influence of various dimensions on farmers' participation in food safety governance

**Figure 1:**
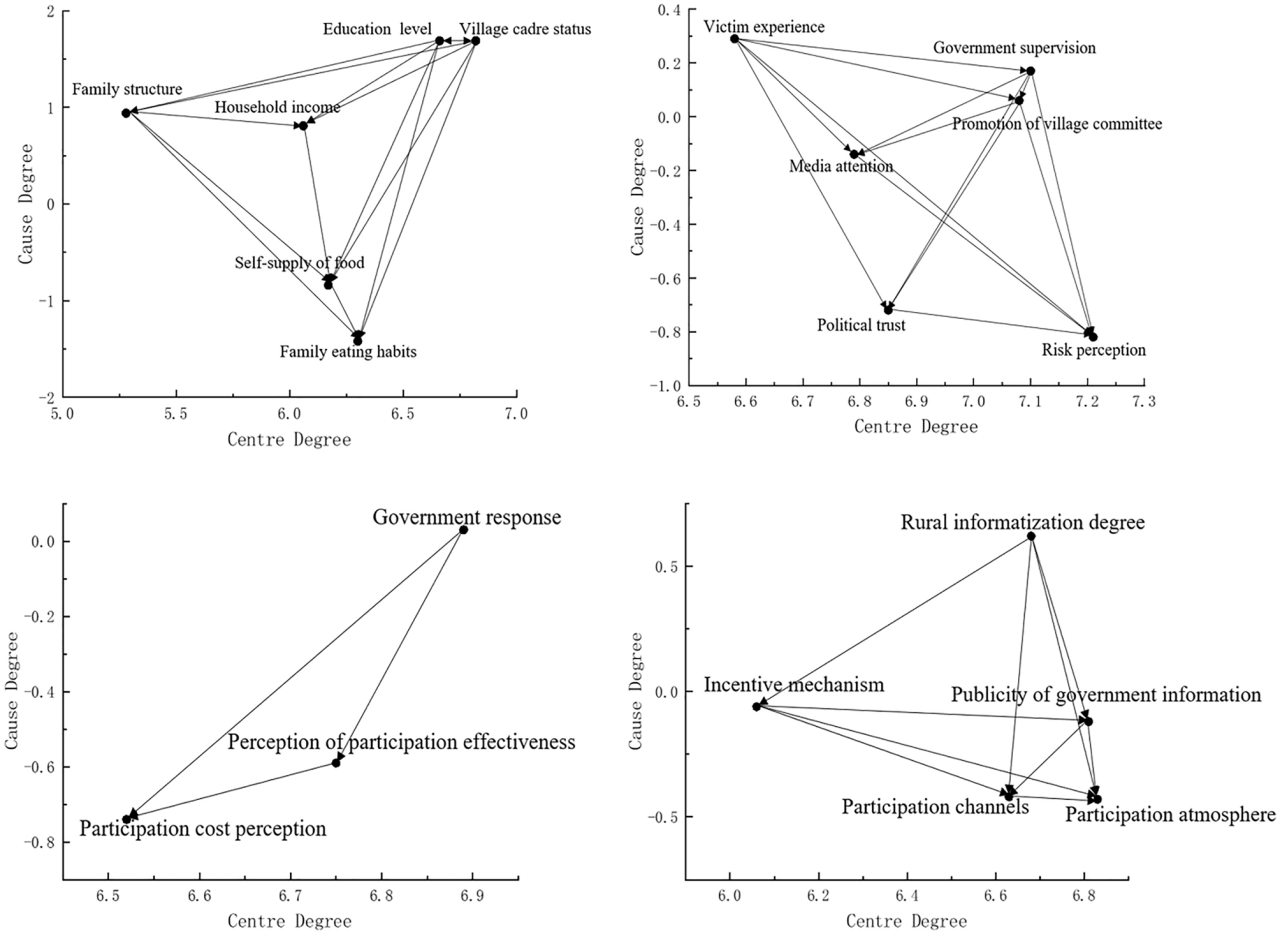
Network diagram of the impact of various dimensions of farmers' participation in food safety governance.

1. Family characteristic dimension. As can be seen from Table 4 and Fig. 1, in terms of centrality, the centrality of the identity of village cadres is higher, followed by the level of education, and the centrality of other factors is relatively low. It shows that the identity of village cadres and the level of education play a core role in the family characteristics that affect farmers' participation in food safety governance. In terms of the degree of cause, the identity of village cadres and the degree of education ranked first, indicating that these two factors are the fundamental factors driving farmers to participate in food safety governance. As the lowest cause in the family dimension and even in the whole system of influencing factors, family dietary consumption habits are most easily affected by other factors. In the work of promoting farmers to participate in food safety governance, we should pay attention to the special attributes of their family food consumption habits.
2. The dimension of participating subject. The centrality of risk cognition factors is the highest, followed by the intensity of government supervision and the promotion of village committees, while the centrality of other factors is lower, indicating farmers' own perception of food safety risks. as well as the external subject behavior promoted by government food safety supervision and village committee has an important impact on farmers' participation behavior. At the same time, the degree of risk cognition is the lowest in this dimension, indicating that farmers' subjective risk cognition is the key factor, and the basic path of "cognition-behavior" should be followed when promoting farmers' participation in food safety governance. The victimization experience has the greatest influence on other factors in terms of cause degree, indicating that the current behavior driving factors of food safety governance subjects are mainly self-interest, lack of self-awareness.
3. The dimension of participation process. The centrality and cause of government response factors rank first, which is obviously different from other factors, indicating that the timeliness and effectiveness of government response is the primary factor to promote farmers' participation in sustainable food safety governance. In addition, the perception of participation cost is most affected by other factors, so reducing participation cost plays an important role in promoting farmers' participation in food safety governance.
4. Participation environment dimension. Participation atmosphere is the most central factor, indicating that participation atmosphere has the strongest overall influence relationship in the participation environment, followed by the degree of government information disclosure, indicating that farmers' participation in food safety governance is very dependent on government information disclosure. From the perspective of cause degree, the degree of rural informatization has the greatest impact on other factors. Therefore, priority must be given to the construction of rural information infrastructure to improve farmers' acceptance and use of information technology. Secondly, participation atmosphere and participation channel, as the two factors with the lowest degree of reason, have strong restriction and promotion, and are the key environmental factors for farmers to participate in food safety governance.

### Hierarchical structure and correlation path of influencing factors of Farmers' participation in Food Safety Governance-ISM

The basic principle of the ISM model is that on the basis of determining the various factors that affect the system and their interrelations, the information is processed to clarify the relevance and hierarchy among the factors, so as to find the main (key) factors and their internal relations^46^. The influencing factors of farmers' participation in food safety governance can not only play an independent role, but also interact with each other. Therefore, this paper will use the ISM model to further analyze the overall correlation path and multi-level structure of the influencing factors.

#### Explain the construction of structural model

1. Establish the adjacency matrix T and the reachability matrix M. On the basis of the data obtained from the comprehensive influence matrix B, Set threshold λ, λ can be calculated through mathematical methods (λ = α + β, α and β is the mean and standard deviation of the elements in the synthesis matrix B)^47^, or determined by decision-makers and experts based on specific actual situations λ^48^. This article sets the threshold λ = 0.19, based on the threshold calculation results and the rationality of the structure presentation, and the adjacency matrix is further constructed as follows:
  4 $${T}_{ij}=\left\{\begin{array}{c}0, \,{b}_{ij}<\lambda \\ 1,\, {b}_{ij}\ge \lambda \end{array}\right.$$

In the formula, it is the constituent element of the comprehensive influence matrix B. the Boolean algorithm is used in the power operation of the matrix. When the formula (5) is satisfied, the reachable matrix M is obtained, the results are detailed in Table 5.

**Table 5:** Accessibility Matrix M for Farmers' participation in Food Safety Governance.

Variables	a1	a2	a3	a4	a5	a6	a7	a8	a9	a10	a11	a12	a13	a14	a15	a16	a17	a18	a19	a20
a1	1	0	0	0	1	1	1	1	1	1	1	1	1	1	1	1	1	1	1	1
a2	0	1	0	0	1	1	1	1	1	1	1	1	1	1	1	1	1	1	1	1
a3	0	0	1	0	1	1	0	1	1	0	0	0	0	0	0	0	0	0	0	0
a4	0	0	0	1	1	0	0	0	1	0	0	0	0	0	0	0	0	0	0	0
a5	0	0	0	0	1	0	0	0	0	0	0	0	0	0	0	0	0	0	0	0
a6	0	0	0	0	0	1	0	0	0	0	0	0	0	0	0	0	0	0	0	0
a7	0	0	0	0	1	1	1	1	1	0	0	0	1	0	0	0	0	0	0	0
a8	0	0	0	0	0	0	0	1	0	0	0	0	0	0	0	0	0	0	0	0
a9	0	0	0	0	1	0	0	0	1	0	0	0	0	0	0	0	0	0	0	0
a10	0	0	0	0	1	0	0	1	1	1	0	0	0	0	0	0	0	0	0	0
a11	0	0	0	0	1	1	0	1	1	1	1	1	1	1	1	1	0	1	1	0
a12	0	0	0	0	1	1	0	1	1	1	1	1	1	1	1	1	0	1	1	0
a13	0	0	0	0	1	0	0	0	1	0	0	0	1	0	0	0	0	0	0	0
a14	0	0	0	0	0	0	0	0	0	0	0	0	0	1	0	0	0	0	0	0
a15	0	0	0	0	1	1	0	1	1	1	1	1	1	1	1	1	0	1	1	0
a16	0	0	0	0	1	0	0	0	1	0	0	0	1	0	0	1	0	0	0	0
a17	0	0	0	0	1	1	0	1	1	1	1	1	1	1	1	1	1	1	1	0
a18	0	0	0	0	1	1	0	1	1	1	1	1	1	1	1	1	0	1	1	0
a19	0	0	0	0	1	0	0	0	1	0	0	0	0	0	0	0	0	0	1	0
a20	0	0	0	0	0	0	0	0	0	0	0	0	0	0	0	0	0	0	0	1

5 $$M={(T+E)}^{n+1}={(T+E)}^{n}\ne {\left(T+E\right)}^{n-1}\ne T+E$$

(2) Determine the reachable set, antecedent set and common set, and divide the hierarchical structure. Based on the reachable matrix M, the reachable set is a set composed of items with row elements of 1 in the matrix, the antecedent set is the set of items with column elements of 1 in the matrix, and the common set is the set of influencing factors of the reachable matrix, that is, the results are detailed in Table 6.

**Table 6:** Reachable set, antecedent set and common set.

Variables	Reachable set(R)	Antecedent set(Q)	Intersection(A = R ∩ Q)
a1	1,5,6,7,8,9,10,11,12,13,14,15,16,17,18,19,20	1	1
a2	2,5,6,7,8,9,10,11,12,13,14,15,16,17,18,19,20	2	2
a3	3,5,6,8,9	3	3
a4	4,5,9	4	4
a5	5	1,2,3,4,5,7,9,10,11,12,13,15,16,17,18,19	5
a6	6	1,2,3,6,7,11,12,15,17,18	6
a7	5,6,7,8,9,13	1,2,7	7
a8	8	1,2,3,7,8,10,11,12,15,17,18	8
a9	5,9	1,2,3,4,7,9,10,11,12,13,15,16,17,18,19	9
a10	5,8,9,10	1,2,10,11,12,15,17,18	10
a11	5,6,8,9,10,11,12,13,14,15,16,18,19	1,2,11,12,15,17,18	18,11,12,15
a12	5,6,8,9,10,11,12,13,14,15,16,18,19	1,2,11,12,15,17,18	18,11,12,15
a13	5,9,13	1,2,7,11,12,13,15,16,17,18	13
a14	14	1,2,11,12,14,15,17,18	14
a15	5,6,8,9,10,11,12,13,14,15,16,18,19	1,2,11,12,15,17,18	18,11,12,15
a16	5,9,13,16	1,2,11,12,15,16,17,18	16
a17	5,6,8,9,10,11,12,13,14,15,16,17,18,19	1,2,17	17
a18	5,6,8,9,10,11,12,13,14,15,16,18,19	1,2,11,12,15,17,18	18,11,12,15
a19	5,9,19	1,2,11,12,15,17,18,19	19
a20	20	1,2,20	20

The hierarchy from the highest level to the lowest level is determined as follows:

6 $$L=\left\{{S}_{i}|P({S}_{i})\cap Q({S}_{i})=C({S}_{i})\right\}$$

**Figure 2:**
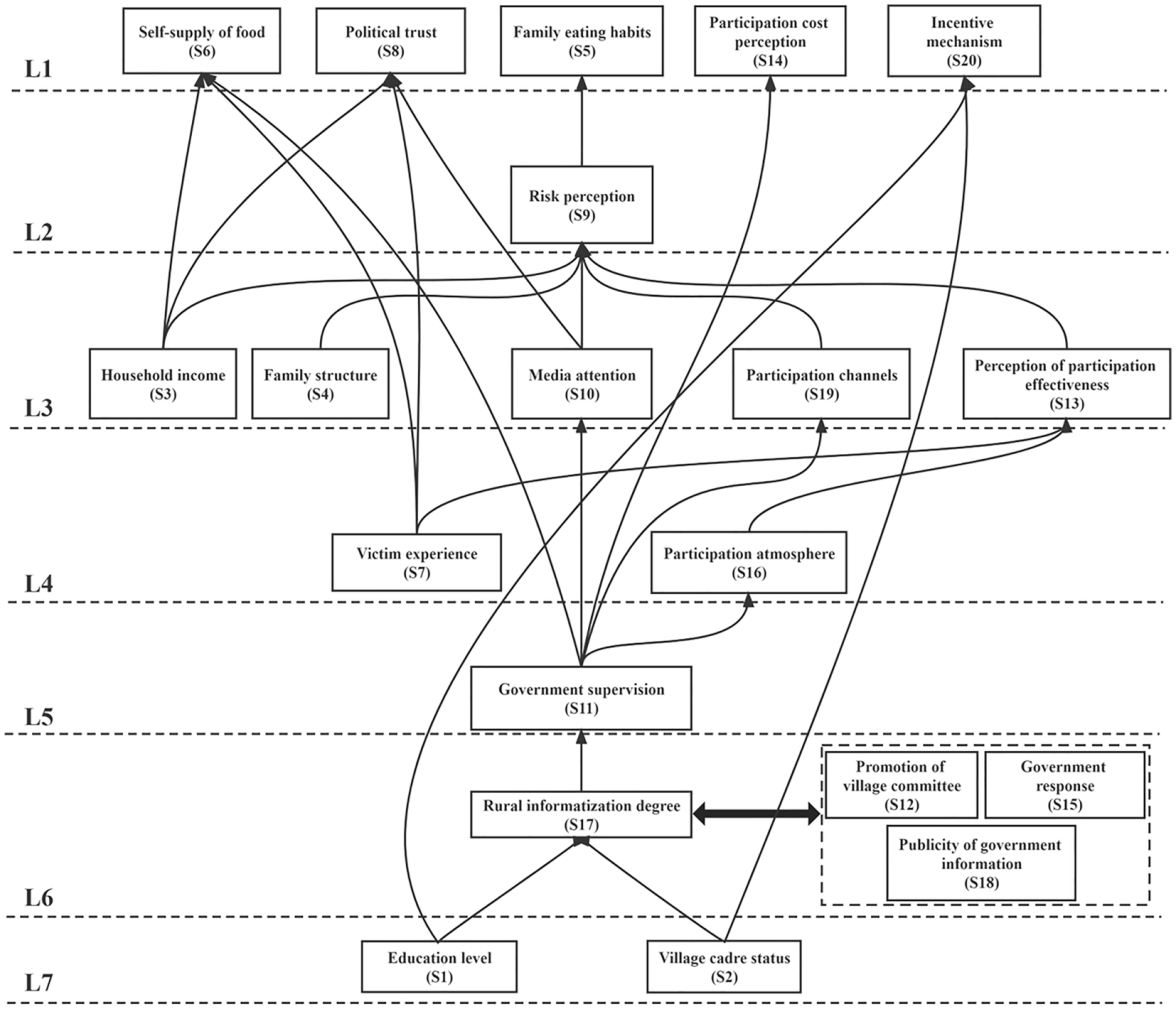
Hierarchy and correlation path of influencing factors.

According to the hierarchical division criterion, the first level division (L1) of the influencing factors can be realized. after the first level is determined, all the row and column factors to which the first level factors belong are deleted, the above steps are continued to divide into the second level, and so on. The multi-level structure of the influencing factors is obtained (as shown in Table 7 and Fig. 2).

**Table 7:** Hierarchical structure of influencing factors.

Hierarchy	Factors
Level 1(Top level)	a5,a6,a8,a14,a20
Level 2	a9
Level 3	a3,a4,a10,a13,a19
Level 4	a7,a16
Level 5	a11,a12,a15,a18
Level 6	a17
Level 7(Bottom level)	a1,a2

#### Hierarchical structure and correlation path Analysis of influencing factors

It can be seen from Fig. 2 that the influencing factors of farmers' participation in food safety governance show a ladder-like distribution structure of seven levels. L5, L6 and L7 represent deep factors, L3 and L4 represent transitional factors, and L1 and L2 represent shallow factors. Among them, education level (S1) and village cadre status (S2) are the deep factors at the bottom, both of which belong to the dimension of family characteristics, but they are independent of each other and have a profound impact on other factors. It is the most fundamental factor to promote farmers' participation in food safety governance. In addition, the intensity of government supervision (S11) is strongly related to the promotion strength of the village committee (S12), the government response (S15) and the degree of disclosure of government information (S18), and the degree of rural informatization (S17) as a deeper factor. It is not only affected by the lower level, but also plays a role in other levels of factors, playing a key node role in the overall hierarchical structure. Among the transitional factors, the murder experience of the fourth level (S7) and the family income (S3) and family structure (S4) of the third level are not affected by the lower level, so it is also beneficial to consider them from the overall perspective. The real role of linking the upper and lower layers are the fourth level of participation atmosphere (S16) and the third level of media attention (S10), participation channels (S10) and participation effectiveness perception (S13). Among the shallow factors, the risk perception of the second layer (S9) is affected by all the factors of the third layer and acts on the household eating and consumption habits of the first layer (S5). In the first layer, food self-sufficiency (S6), political trust (S8), household dietary consumption habits (S5), participation cost perception (S14) and incentive mechanism (S20) all directly affect farmers' participation in food safety governance. It is the most direct factor to promote farmers' participation in food safety governance.

In the hierarchical structure of the influencing factors of farmers' participation in food safety governance. The path with the most related factors is "education level (S1) /village cadre identity (S2)-rural informatization level (S17)-government supervision (S11)/village committee promotion (S12) /government response (S15)/government information disclosure (S18)-participation atmosphere (S16)-participation effectiveness perception (S13)-risk awareness (S9)-family dietary consumption habits (S5)." The path with the least related factors is "education level (S1) /village cadre identity (S2)-reward mechanism (S20)".

### Attribution of factors affecting Farmers' participation in Food Safety Governance– MICMAC

#### Dependence and driving force calculation

Based on the hierarchical division of the influencing factors based on the ISM model, the MICMAC analysis method is used to classify the attributes by calculating the dependence and driving forces of the influencing factors, which is beneficial to the in-depth analysis of the status and function of the influencing factors, and puts forward the corresponding countermeasures and suggestions.

On the basis of the reachable matrix M, the dependence and driving force of the influencing factors are determined. Dependence refers to the influence of other factors on this factor, which is the sum of the column elements of the reachable matrix M, and the driving force refers to the influence of this factor on other factors, which is the sum of the row elements of the reachable matrix M. the results are detailed in Table 7.

7 $${X}_{j}=\sum_{i=1}^{xx}{m}_{ij},j=1, 2, \cdots , 19$$

8 $${Y}_{i}=\sum_{j=1}^{xx}{m}_{ij}, i=1, 2, \cdots , 19$$

In formula (7), (8), it represents the constituent elements of the reachable matrix M.

**Figure 3:**
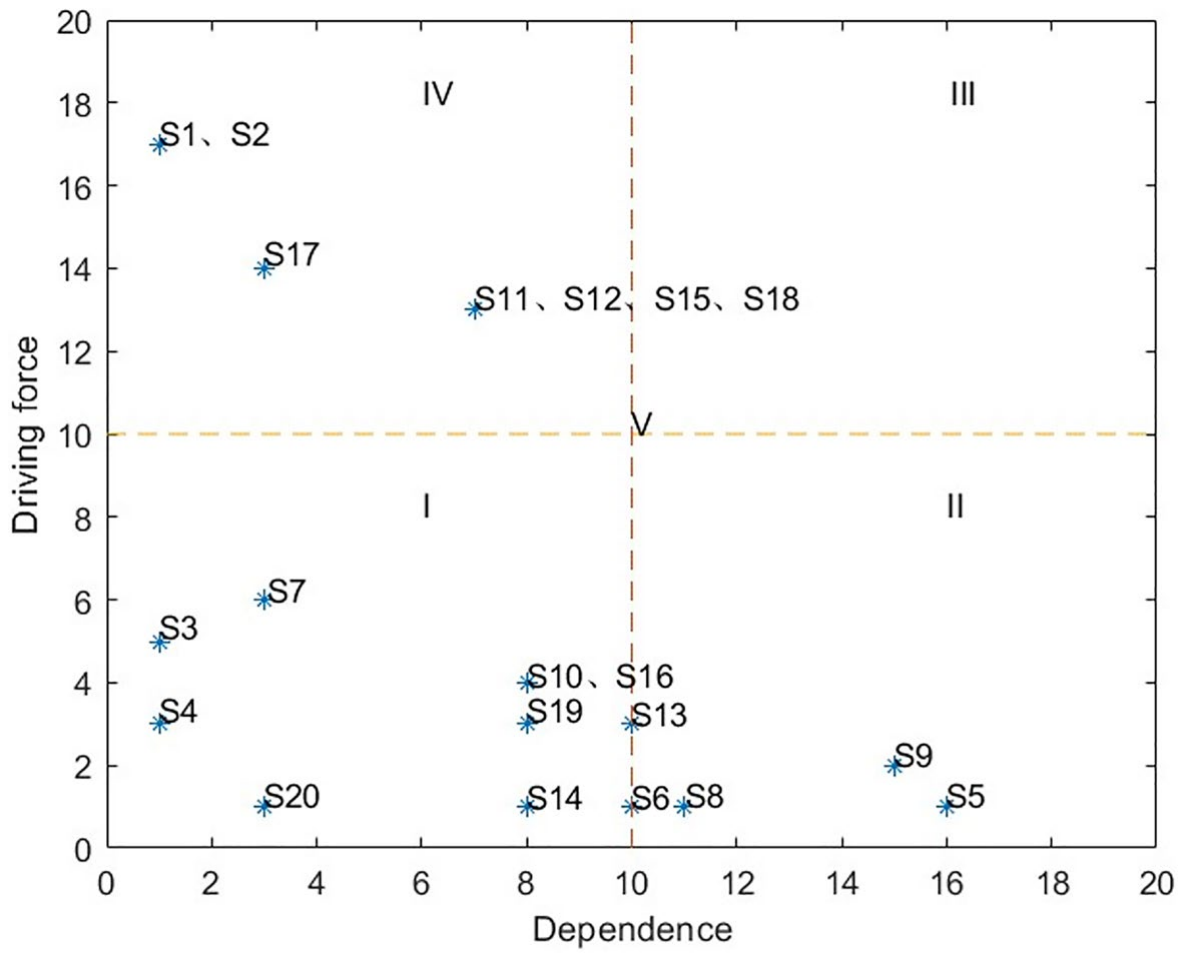
Attribute classification diagram of influencing factors.

Based on the dependence and driving force values of the influencing factors in Table 8, the attribute division map of influencing factors is constructed in the form of two-dimensional coordinate axis (as shown in Fig. 3), which can be divided into four quadrants and a region (distinguished by I, II, III, IV, V). The factor attributes in the quadrant are independent factors, dependent factors, related factors, independent factors and adjustment factors.

**Table 8:** Dependence and driving forces of influencing factors.

Factors	Dependence	Driving force	Factors	Dependence	Driving force
S1	1	17	S11	7	13
S2	1	17	S12	7	13
S3	1	5	S13	10	3
S4	1	3	S14	8	1
S5	16	1	S15	7	13
S6	10	1	S16	8	4
S7	3	6	S17	3	14
S8	11	1	S18	7	13
S9	15	2	S19	8	3
S10	8	4	S20	3	1

#### Analysis of the result of attribute division of influencing factors

Autonomous factors (I quadrant), including family income (S3), family structure (S4), experience of murder (S7), media attention (S10), participation cost perception (S14), participation atmosphere (S16), participation channel (S19) and incentive mechanism (S20). The dependence and driving force of autonomous factors are weak, and the link with other influencing factors in the whole system is simple and less related. Dependent factors (II quadrant), including household eating habits (S5), political trust (S8) and risk perception (S9), are more controlled by other factors, while the driving force is weaker. Based on the ISM hierarchical structure chart, although the above three factors are located in the shallow layers of L1 and L2, they are more influenced by other deep factors than those at the same level and are the most direct factors affecting farmers' participation in food safety governance. Therefore, formulating relevant policies around these three factors may have more obvious effects and have a special role in farmers' participation in food safety governance, which should be given special attention. The correlation factor (III quadrant) is characterized by high dependence and high driving force. Independent factors (IV quadrant), including education (S1), village cadre status (S2), government supervision (S11), village committee promotion (S12), government response (S15), rural informatization (S17) and government information disclosure (S18). It has the characteristics of high driving force and low dependence, which is less affected by other factors, but has greater influence on other factors. Based on the ISM hierarchical structure chart, the above factors are located at the deep level of L5, L6, and L7, with a simple chain structure. However, they play a core driving role in the entire system and are the deep core factors that promote farmers' participation in food safety governance. Among them, education level (S1) and village cadre identity (S2) are the fundamental characteristic factors, which are related to individual farmers. Adjusting factors (area V), including food self-sufficiency (S6) and participation efficacy perception (S13), it can be seen from the chart that the above factors have mean level dependence and low driving force, and their effects are between autonomous factors and dependent factors.

It is worth noting that, based on the ISM hierarchical structure diagram, MICMAC is further adopted for factor attribute partitioning, which may lead to information loss issues in the process of simplifying complex systems. The results show that generally speaking, the deeper the level of factors, the greater the driving force and the smaller the dependency. The shallower the level of factors, the smaller the driving force and the greater the dependency. However, factors that are not particularly dependent and driven do not necessarily mean they are not important, such as the reward mechanism (S20) mentioned in the article. As the factor with the lowest dependence and driving force, this factor is located at the shallowest level of the hierarchical structure, but is associated with the deepest level of education (S1) and village cadre identity (S2). Specifically, different reward mechanisms should be adopted for farmers with different levels of education and whether they have the status of village cadres, such as reporting bonuses or reputation rewards, which are also enlightening for policy formulation. However, this article mainly focuses on the level of the entire system, based on the hierarchical structure of ISM, and through the attribute division of MICMAC, aiming to identify factors that have more systematic contributions or can have a greater impact on policy effectiveness.

## Conclusions and suggestions

Through the DEMATEL-ISM-MICMAC combination model, this paper studies the influencing factors and related paths of farmers' participation in food governance, and draws the following conclusions:

The main results are as follows:

1. The influence of family characteristics, participants, participation process and participation environment on farmers' participation in food safety governance can be characterized by the relationship network of specific factors in each dimension. The identity of village cadres and the level of education are in the core position of the family characteristics, and have the greatest impact on other factors, which is the core factor. Risk perception is the basic factor of the participant dimension, which is the most affected and needs to be paid more attention. Government response has the highest degree of cause and centrality in the dimension of participation process, which is the most important factor to ensure the effectiveness and sustainability of the process. The degree of rural informatization, as the factor with the highest degree of cause in the participation environment, is a necessary prerequisite for other environmental factors to play a role, and the participation atmosphere has the characteristics of the highest degree of centrality and the lowest degree of cause, which is the key factor in this dimension.
2. The influencing factor system of farmers' participation in food safety governance consists of seven levels, and the key factors can be extracted by combining the attribute division of dependence-driving force. The level of education and the identity of village cadres are at the bottom of the hierarchical structure, and their driving forces are the strongest, which are the fundamental characteristic factors for farmers to participate in food safety governance. The degree of rural informatization and the intensity of government supervision, the promotion of village committees, the government response and the disclosure of government information are located at the sixth and fifth levels respectively, with a strong driving force. it is the deep core factor to promote farmers' participation in food safety governance. The excessive factor layer, including the murder experience of the fourth layer, the atmosphere of participation, and the third layer of family income, family structure, media attention, participation efficacy perception and participation channels, all have the characteristics of weak driving force. Among the shallow factors, risk cognition is located in the second layer, which is affected by the joint effect of the next layer; food self-sufficiency, political trust, family eating habits, participation cost perception and reward mechanism are located in the first layer, which are the direct influencing factors of farmers' participation in food safety governance, among which risk cognition, political trust and family eating habit dependence are the highest, which should be treated as special factors.

Based on the above research results, the following countermeasures and suggestions are put forward: (1) To ensure the supply of educational resources in rural areas, improve the per capita education level in rural areas, and bring food safety education into the curriculum of primary and middle school students, supplemented by daily propaganda in various forms and channels to enhance the food safety literacy and risk awareness of rural residents. (2) To link the effectiveness of rural food safety governance with the performance of village cadres, make good use of the reward and punishment mechanism to consolidate the responsibility of village cadres, give full play to the role promoted by village committees, and speed up the formation of an atmosphere for participation in rural food safety governance. (3) The core of food safety governance lies in information symmetry, so it is necessary to improve the degree of informatization in rural areas from many aspects, such as the construction of information infrastructure, the construction of information sharing network and the reduction of the threshold for the use of information technology. (4) The territorial government should adhere to the principle of "people-oriented", rely on the digital platform to innovate the way of food safety supervision, broaden the channels of opinion expression, strengthen the two-way supply of food safety information with farmers, and effectively improve the level of political trust of farmers, in order to promote farmers to participate in food safety governance.

## Discussion

This paper focuses on constructing the system of influencing factors of farmers' participation in food safety governance, revealing the relationship network of influencing factors, establishing the hierarchical structure and correlation path of influencing factors, and classifying them according to their dependence-driving force values, the attributes of the factors according to their dependency-driver values, which helps to clarify the relationships between the factors and identify the deep core factors and special influcing factors. The marginal contribution of this article lies in: Firstly, it enriches the research scope of the influencing factors of farmers' participation in food safety governance. Based on a systematic perspective, the influencing factors of farmers' participation in food safety governance are divided into four dimensions: family characteristics, participants, participation process, and participation environment, and internal and external factors are comprehensively considered as much as possible; Secondly, using the combination method of DEMATEL-ISM-MICMAC and comparing existing literature, further exploration was conducted on the influencing factors of farmers' participation in food safety governance, which can provide more targeted reference for food safety governance decision-making departments to formulate policies to promote farmers' participation in food safety governance; Thirdly, this article chooses family characteristics as a substitute for the commonly set personal characteristics dimension in existing research, which is closer to the household based food consumption structure in rural China and increases the possibility of farmers participating in food safety governance among the influencing factors of the entire system.

Compare the research findings of this article with existing research. Education level and the identity of village cadres are fundamental characteristic factors, which are basically consistent with the research conclusions of Feng Mei et al. The higher the education level of the respondents or being a member of the Communist Party of China, the more likely they are to significantly increase the probability of public participation in food safety supervision^49^. Similar to the research conclusions of Wu Linhai et al., village committees perform best in assisting in reporting and investigating the illegal acquisition of sick and dead livestock and poultry (such as sick and dead pigs)^22^, indicating that farmers may increase their participation in food safety governance due to their own level of knowledge or political identity. The deep core factors include the level of rural informatization, government supervision, the promotion of village committees, government response, and the degree of government information disclosure. Similar to the research conclusions of Zhang Bei et al.^1^, Wu Linhai et al.^22,37^, Zhang Ligang et al.^50^, Xiong Chunlin et al.^51^, combined with the ISM hierarchical structure chart, the common feature of the deep influencing factors and existing research results is that the level of rural informatization drives other factors, recognized that the improvement of rural informatization level can provide sufficient support to the government and village committees in the field of food safety governance at the technical level. As special factors, risk perception, political trust and family eating habits need to be specifically discussed. The first two factors are referential to the research conclusions of Wang Jianhua et al.^8^, Xi Yunxiao et al.^31^. In combination with the Theory of planned behavior, food safety risk perception will reverse affect food safety participation behavior, but there are also contradictions. There are also many specific influencing factors that lead to deviation, including political trust. The political trust of urban and rural residents has a significant negative impact on their perception of food safety risks, and the influencing factors of the conflict between farmers' risk perception and governance behavior can serve as an extension of the research perspective in the future. However, no similar literature has been found regarding the factor of household dietary consumption habits. The consideration for inclusion in the indicator system is that with the rapid development of urbanization in China, the phenomenon of abandoned agricultural, forestry, farmland, and pastoral cultivation is gradually emerging, and the self-sufficient "safe food" provided by farmers is decreasing. Moreover, the food source channels for rural residents are becoming increasingly diverse, and the dietary structure is becoming more complex and diverse. The consumption proportion of processed food in workshops, as well as non staple food such as forestry products and marine products, continues to increase. When the rural food safety supervision system is not sound, household dietary consumption habits may become an important influencing factor affecting farmers' participation in food safety governance. Therefore, in the future, we can also conduct further research on whether and how household dietary consumption habits affect farmers' participation in food safety governance.

Similarly, this article also has some limitations: Firstly, while DEMATEL has a certain degree of authority, it inevitably has subjectivity, and there are still many shortcomings in the data description compared to traditional econometric regression. Through literature reference, many scholars have adopted the combination method of LOGIT ISM for research. In their research, LOGIT, as the method of the first stage, includes public data^52^ and self research data^53^, which can provide statistical description of the data. LOGIT can provide reliable Empirical evidence for ISM. However, at the same time, they are unable to achieve an effective connection between LOGIT and ISM, and it is still necessary to subjectively determine and rank the influencing factors by experts based on the significance of LOGIT results. Therefore, as a method of systematic research, there is still room for exploration on how to scientifically and effectively integrate ISM with research topics. Secondly, the indicators selected by the influencing factor system are based on the existing achievements at home and abroad, and cannot be complete in all aspects. There may be some deviation from the actual situation. In the future, the current deficiency can be made up by clarifying expert review standards, expanding expert sample size and selecting precise indicators, so as to make the results more representative and scientific.

## Supplementary Information

Supplementary Information.

## Data Availability

All data generated or analysed during this study are included in this published article [and its Supplementary Information files].
